# Music for a Brighter World: Brightness Judgment Bias by Musical Emotion

**DOI:** 10.1371/journal.pone.0148959

**Published:** 2016-02-10

**Authors:** Joydeep Bhattacharya, Job P. Lindsen

**Affiliations:** Department of Psychology, Goldsmiths, University of London, London, United Kingdom; University of Bologna, ITALY

## Abstract

A prevalent conceptual metaphor is the association of the concepts of good and evil with brightness and darkness, respectively. Music cognition, like metaphor, is possibly embodied, yet no study has addressed the question whether musical emotion can modulate brightness judgment in a metaphor consistent fashion. In three separate experiments, participants judged the brightness of a grey square that was presented after a short excerpt of emotional music. The results of Experiment 1 showed that short musical excerpts are effective emotional primes that cross-modally influence brightness judgment of visual stimuli. Grey squares were consistently judged as brighter after listening to music with a positive valence, as compared to music with a negative valence. The results of Experiment 2 revealed that the bias in brightness judgment does not require an active evaluation of the emotional content of the music. By applying a different experimental procedure in Experiment 3, we showed that this brightness judgment bias is indeed a robust effect. Altogether, our findings demonstrate a powerful role of musical emotion in biasing brightness judgment and that this bias is aligned with the metaphor viewpoint.

## Introduction

Throughout history and across cultures, the concepts of good and evil are associated with brightness and darkness, respectively. Examples of such associations can be found in everyday phrases like *‘look on the bright side’* and *‘the forces of darkness’*, depictions of heaven and hell, literary descriptions of happiness and depression, and rituals and attire related to weddings and funerals. This linking of one idea to another is known as a conceptual metaphor, which is thought to aid in understanding and communicating abstract concepts (e.g. good or evil) by applying knowledge of more concrete or physical concepts (e.g. brightness or darkness) [1].

Persistent metaphorical associations such as these have been shown to affect cognition and sensory experiences (see for a review [2]). For example, smiling faces appear to be brighter than frowning faces [3]), grey squares are perceived as brighter after evaluating positive words than negative words [4], or remembering an ethical deed make the immediate surroundings appear brighter than remembering an unethical deed [5] (but see also [6]). These findings suggest that metaphors link brightness judgment to emotion.

A stimulus that has been successfully and reliably used to induce emotions and affective states is music [7]. More than 2500 years ago, the celebrated Chinese philosopher Confucius remarked, "Music produces a kind of pleasure which human nature cannot do without." Human civilizations have undergone a sea change since then. Yet after two millennia the statement stands as bold and true as ever. We spend billions of dollars annually on music, and in every society, music is experienced and consumed by most people in their everyday lives and in diverse situations [8, 9]. A considerable amount of music experience is deliberate [10], and emotions appear prominently in people’s reported motives for listening to music [11]. The experience of music listening is mediated by many factors including musical and cultural background [12], musical preferences [13], familiarity [14], personality [15], and the social context [16]. Yet there is recent evidence that certain basic emotions, like happy and sad, expressed by musical stimuli are universally recognized [17]. There could be a systematic relationship between musical profiles (e.g., crescendos) and psychophysical autonomic responses [18], which are not necessarily under listener’s cognitive control.

Therefore, it is not too surprising that recent research shows that musical emotion could alter our decision making processes (see for a review, [19]). For example, ratings of the taste of wine reflected the emotional connotation of the background music [20]. The emotional judgment of facial stimuli could be biased towards the direction of the emotional valences of the musical primes [21] or emotional judgment of complex visual stimuli were biased towards the direction of emotional arousals of the musical primes [22]. Further, Riener and colleagues [23] showed that when participants were led to a music-induced sad mood, while standing at the bottom of a steep hill, they tend to overestimate the steepness of a hill in a similar manner to those made by participants under physical stress [24]. Furthermore, in an affective priming paradigm using consonant and dissonant chords as priming stimuli and emotional words as target stimuli, Sollberger and colleagues [25] showed that the target words were evaluated faster and more accurately for affectively congruent prime-target pairs than for incongruent ones.

However, it is not yet known whether musical emotion can modulate brightness judgment in a metaphor consistent fashion. Hence, we investigated the effect of musical emotion on brightness judgment. In three separate experiments, participants judged the brightness of a grey square that was presented after an excerpt of emotional music. Although in previous research music was often used as a background stimuli, and the exposure to music was relatively long (in the order of minutes), we assumed that even relatively short (in the order of seconds) musical excerpts can be used as effective emotional primes that could lead to influence brightness judgment. Based on the prevalent metaphorical mapping (positive = bright; negative = dark), we predicted that the emotional valence of the musical excerpt systematically affects brightness judgement, i.e. a positive music excerpt biases judgment towards brighter while a negative music excerpt biases judgment towards darker.

## Materials and Methods

### Ethics Statement

All participants gave written informed consent before the start of the experiment. The experimental protocol followed the guidelines of the declaration of the Helsinki and was approved by the Ethics Committee of the Department of Psychology at Goldsmiths.

### Stimuli

For all three experiments, we used 56 short musical excerpts as primes that were previously validated by Vieillard and colleagues [26] conveying four target emotions: happiness, sadness, scary, and peacefulness. These four different types of musical primes could be discriminated along the two principal dimensions of Russell's circumplex model of affect [27]: arousal and valence. Musical excerpts with a high rating on both valence and arousal were labelled as happy, a low rating on both valence and arousal was taken to indicate sadness, a high rating on valence combined with a low rating on arousal was considered to indicate peacefulness, and finally, a low rating on valence combined with a high arousal rating was thought to indicate scariness. There were 14 musical excerpts in each emotional category. The length of the segments was between 9 s and 17 s and was matched across categories. The primes were generated as MIDI and synthesized using a piano timbre.

### Analysis of Musical Stimuli

As two auditory attributes, pitch and acoustic brightness, are conceptually related with the visual brightness, we performed two types of analysis of the musical primes. For pitch, we performed a midi-based analysis by the function readmidi.m available at kenschutte.com/midi. For acoustic brightness, we performed an acoustic analysis by the function mirbrightness.m available in the MIR Toolbox [28].

### Experiment 1

#### Participants

Twenty adults (11 females, mean age 27 years) with normal or corrected vision and normal hearing volunteered to take part in Experiment 1. None of the participants were professional musicians or music students.

#### Experimental Procedure

On each of the 56 trials, participants were presented with two grey squares with identical dimensions, background and locations, and a musical prime. The first square was presented for 1 s, after which it was replaced by a fixation cross and the musical prime was played via headphones. After the musical excerpt was finished, participants were asked to rate the valence and arousal of the musical prime on two 7-point Likert scales (valence scale from 1: very unpleasant to 7: very pleasant; arousal scale from 1: very relaxing to 7: very stimulating) presented sequentially. Immediately afterwards, a second grey square was presented for 1 s. The participants were asked to judge whether the second square was brighter or darker than the first by a left or right key press. In the instructions, the participants were told that the change in brightness between the two squares was small but detectable (similar instruction as in [4]), while in reality the same square was presented twice. The order of the musical primes was randomized over participants.

#### Data Analysis

A brightness judgment bias was calculated as the deviation of the average proportion of brighter/darker judgements from 0.5. Positive (negative) deviation from 0.5 would indicate that the second square was judged as brighter (darker) than the first one. The brightness judgment bias was analysed with a 2 (*valence*: positive vs. negative) x 2 (*arousal*: high vs. low) within-subjects ANOVA. This ANOVA was repeated twice; once based on the original classification of the primes by Vieillard and colleagues [26], and once based on the subjective ratings of valence and arousal of each participant in this experiment. The latter classification was done by using a median split on the individual ratings. Effects were considered statistically significant at *p* < 0.05. Further, all reported effect sizes (*r*) were calculated after Rosenthal [29, 30] as follows: $r=\sqrt{t^{2}/\left(t^{2}+df\right)}$. The *r* values were interpreted after Cohen [31] as follows: small effect, *r* = 0.10; medium effect, *r* = 0.30; large effect, *r* = 0.50.

#### Results and Discussion

**Fig 1:**
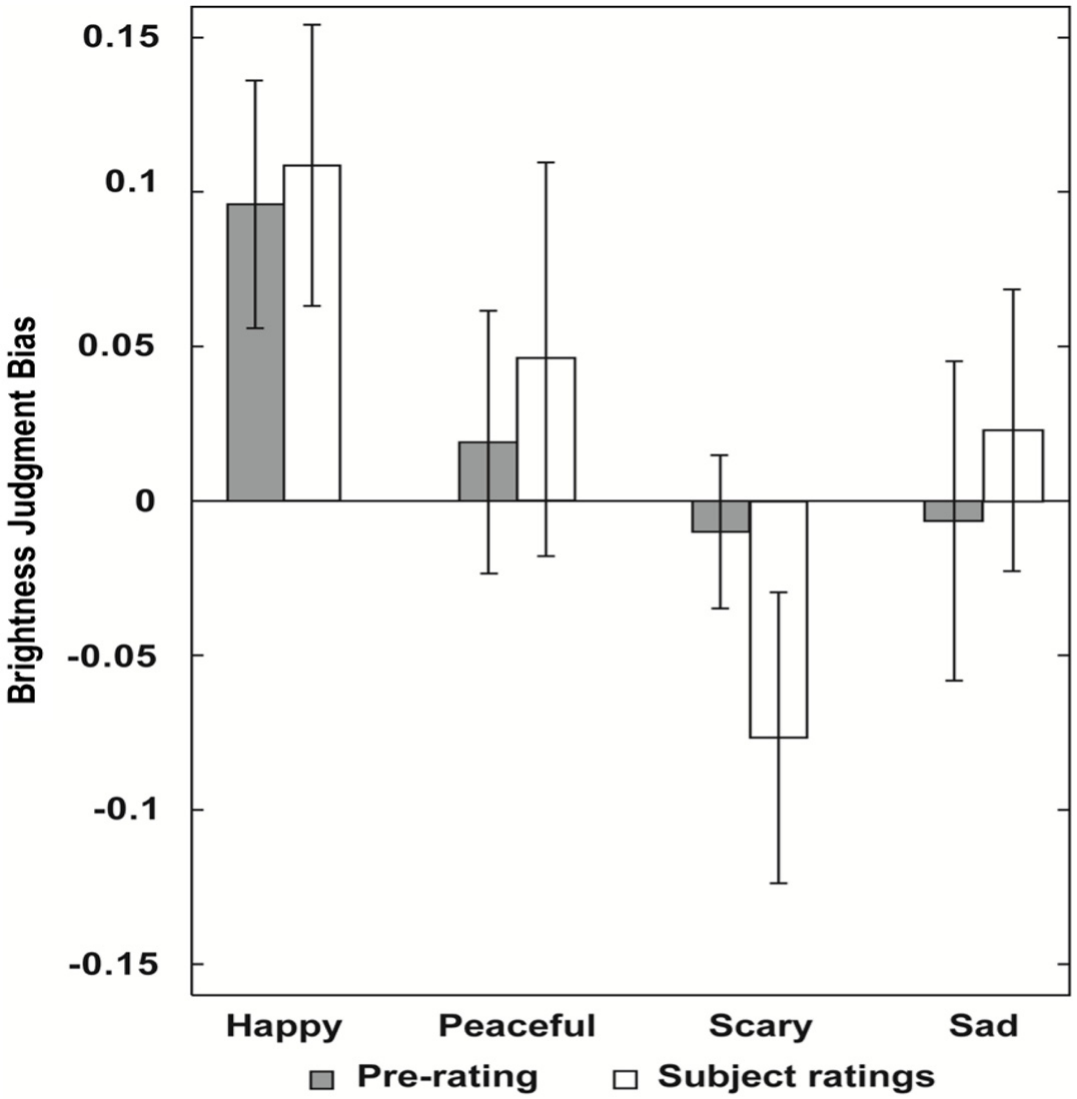
Results of Experiment 1. Brightness judgment biases, separately for happy, peaceful, scary, and sad excerpts. Excerpts were categorized based on the objective intended emotion after [26] or based on the subjective ratings of each participant. Error bars indicate SE of the mean.

The second grey square was judged to be brighter more often following musical primes with positive valence, as compared to primes with negative valence, *F*(1,19) = 5.65, *p* < .03. The effect size was *r* = 0.48. This effect was mostly driven by the happy musical primes (Fig 1). There was no significant effect of arousal, *F*(1,19) = 0.97, *p* = .34, and no significant interaction between valence and arousal, *F*(1,19) = 2.71, *p* = .12.

When the musical primes were categorized based on the subjective ratings of our participants, we observed a similar pattern (Fig 1). The grey squares were judged as brighter after musical primes were subjectively rated as positive valence, *F*(1,19) = 4.67, *p* < .05. The effect size was *r* = 0.44. No significant effect of subjectively rated arousal was observed, *F*(1,19) = 1.02, *p* = .33, and the interaction between valence and arousal was marginal, *F*(1,19) = 3.38, *p* = 0.08.

**Fig 2:**
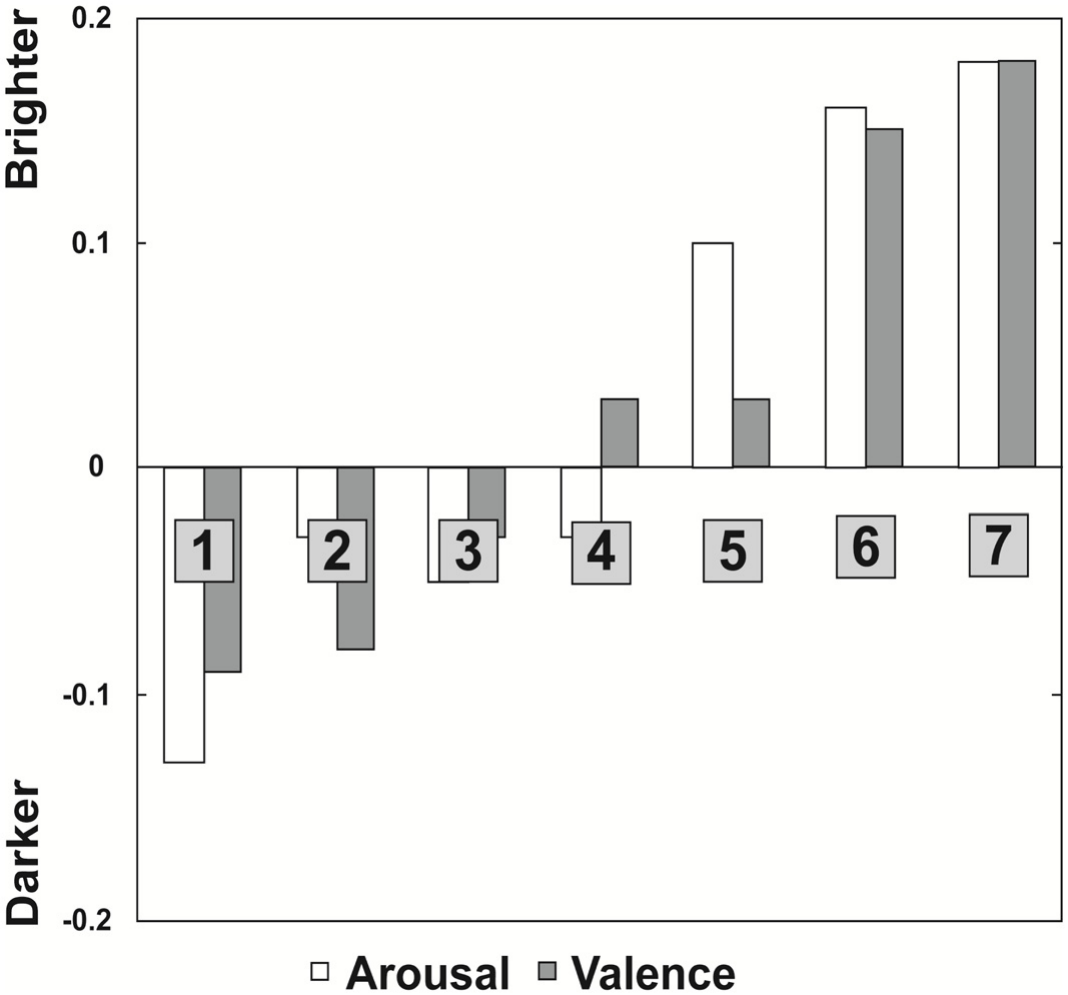
Brightness judgment bias of Experiment 1. Average brightness judgment bias separately for every level on the 7-point Likert scales of Valence and Arousal.

To further investigate the relation between subjective ratings of valence and arousal, and the bias in judged brightness, the average brightness judgment bias was calculated separately for every level on the 7-point Likert scales (Fig 2). Across participants, we observed a linear relationship: the higher the ratings on arousal and valence of musical primes, the higher the bias in brightness judgment. Further, this bias was most conspicuous at the extreme positive end of the scales, i.e. high values of arousal and valence.

It should be noted here that on average the ratings of valence and arousal from our participants are in good agreement with the original classification into the four emotional categories as in [26]; see Table 1.

**Table 1:** Average valence and arousal ratings of musical excerpts. The table lists the average valence and arousal ratings for the four different types of musical excerpts (happy, sad, scary and peaceful) used as primes. (SE of the mean).

Emotional Category	Valence	Arousal
**Happy**	4.73 (.10)	4.86 (.09)
**Sad**	4.05 (.11)	3.86 (.11)
**Scary**	3.57 (.11)	4.30 (.10)
**Peaceful**	4.39 (.09)	3.82 (.10)

The results of Experiment 1 show that happy music makes a subsequently presented grey square to be judged brighter than the identical grey square presented earlier. Further, the brightness judgment bias remains robust irrespective of whether the musical excerpts were pre-categorized as happy or subjectively perceived to be happy. Our results are in accordance with previous studies suggesting that metaphors link brightness judgment to emotion [3–5].

In Experiment 1, musical primes were affectively evaluated before participants provided a brightness judgment. In order to investigate whether such explicit affective evaluation of the musical primes was necessary for the brightness judgment bias to occur, we performed a second experiment which was similar to Experiment 1 but without any emotional evaluation of musical primes.

### Experiment 2

#### Participants

Twenty human adults (16 females, mean age 27 years) with normal or corrected vision and normal hearing volunteered to take part in Experiment 2. None of the participants had taken part in Experiment 1 and none were professional musicians or music students.

#### Experimental Procedure

The same procedure as in Experiment 1 was used, but without the affective evaluation of musical primes, i.e. the ratings of valence and arousal. Participants were shown the second grey square immediately after the music finished and there was no explicit affective evaluation of the musical primes. It should be stressed here that our participants were naive to the experimental aim(s) and the word 'emotion' did not appear on the information sheet given to them prior to the experiment in order to minimize any explicit affective evaluation of the musical excerpts.

#### Results and Discussion

**Fig 3:**
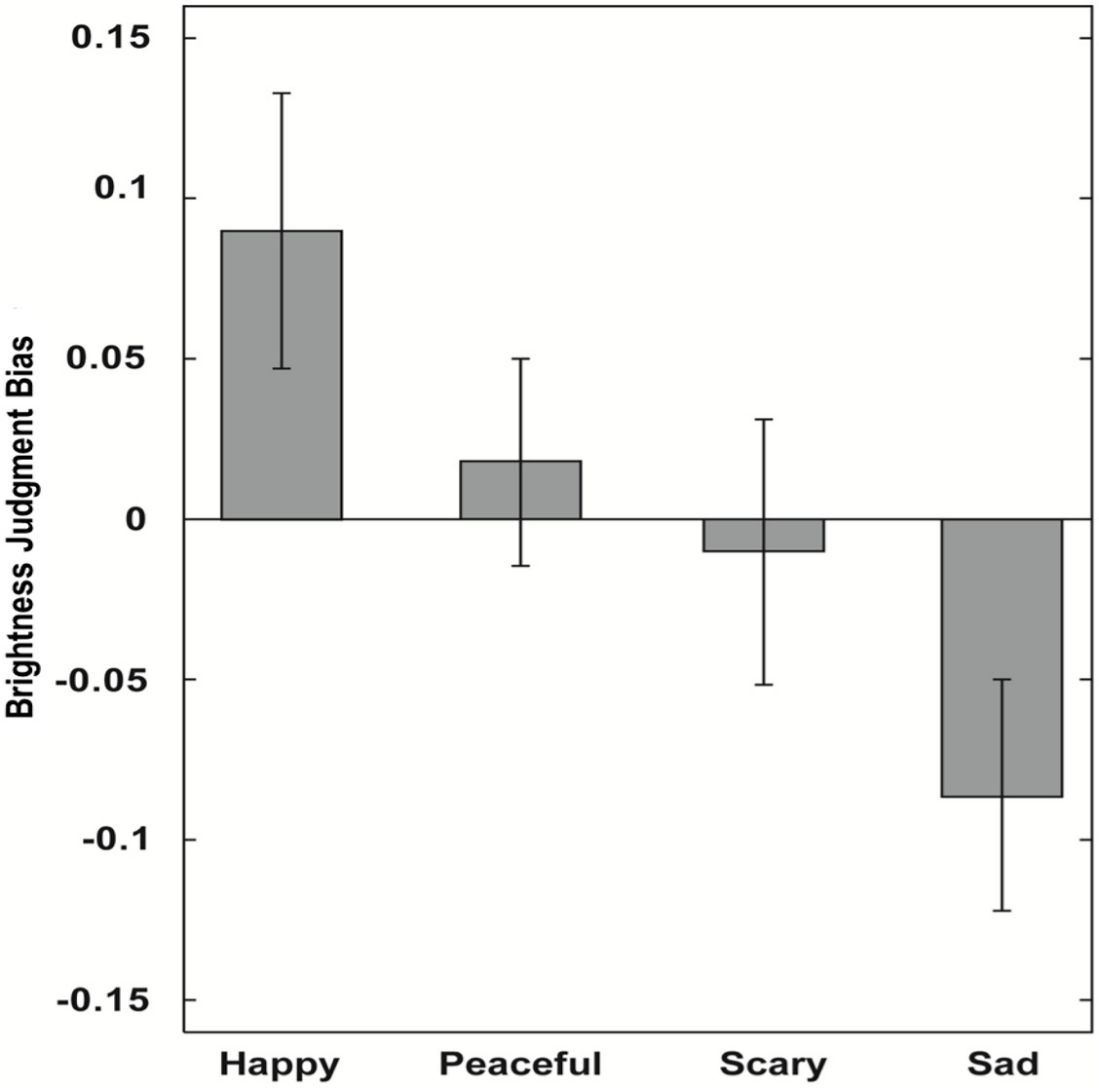
Results of Experiment 2. Brightness judgment biases, separately for happy, peaceful, scary, and sad excerpts. Error bars indicate SE of the mean.

The second grey square was judged to be brighter more often following musical primes pre-categorized as having positive valence, as compared to primes having negative valence, *F*(1,19) = 7.67, *p* < .025. The effect size was *r* = .54. Similar to the results of Experiment 1, happy musical primes biased judgment towards increased brightness. In contrast to the previous results, sad primes clearly biased judgment in the opposite direction towards decreased brightness (Fig 3). The effect of arousal was marginally significant, *F*(1,19) = 4.04, *p* = .059 and the effect size was medium, *r* = 0.42 There was no significant interaction between valence and arousal, *F*(1,19) = 0.01, *p* = .94.

The results of Experiment 2 mostly replicate those of Experiment 1: valence of a musical prime affects brightness judgment. Listening to short segments of happy music makes a grey square being judged brighter, while listening to sad music makes it to be judged darker. The explicit evaluation of the emotional content of the primes is in fact not necessary for this brightness judgment bias to occur, therefore, suggesting that the music induced brightness judgment bias may be an automatic effect. Merely listening to a short excerpt of happy or sad music affects subsequent brightness judgment.

In both Experiments 1 and 2, participants were presented with two grey squares, one before and one after the presentation of the musical prime, and they judged the brightness of the second grey square relative to the brightness of the first square. In order to investigate whether the brightness judgment bias is more related with the memory representation of the first grey square or with the brightness judgment of the second grey square, we performed a third experiment in which the participants rated a grey square presented after musical prime on an absolute scale.

### Experiment 3

#### Participants

Twenty human adults (13 females, mean age 21 years) with normal or corrected vision and normal hearing volunteered to take part in Experiment 3. No participants took part in the previous two experiments.

#### Experimental Procedure

There were two phases in this experiment. In the first phase, the participants had to learn a grey scale with brightness varying from 1 (completely black) to 100 (completely white). In this learning phase, they were presented with squares of varying shades and were asked to judge a brightness rating between 1 and 100 on the grey scale they had previously seen. Participants received immediate feedback on their performance. Adequate learning was identified when performance in the last 10 trails was reasonably close (within ± 10 points) to true luminance level with a minimum of 30 trials. Once the learning criterion was met, in the second phase of this experiment participants were presented with musical primes, and only one square (but with shades varying across trials) was presented on each trial (56 in total) immediately after the musical prime was finished. The participants were asked to judge the brightness of the current square on the grey scale they had learnt earlier. Across trials, the brightness of the squares varied between 20 and 80 to allow for a bias towards both extremes of the spectrum. The order of the musical primes was randomized over participants.

#### Data Analysis

A brightness judgment bias was calculated as the difference between the real level of brightness on the grey scale and the judged level of brightness as reported by the participants. At participant level, this bias value was normalized with respect to the overall mean bias score across all four musical primes. Positive scores indicate a bias towards the brighter judgment, while negative scores indicate a bias towards the darker judgment.

#### Results and Discussion

**Fig 4:**
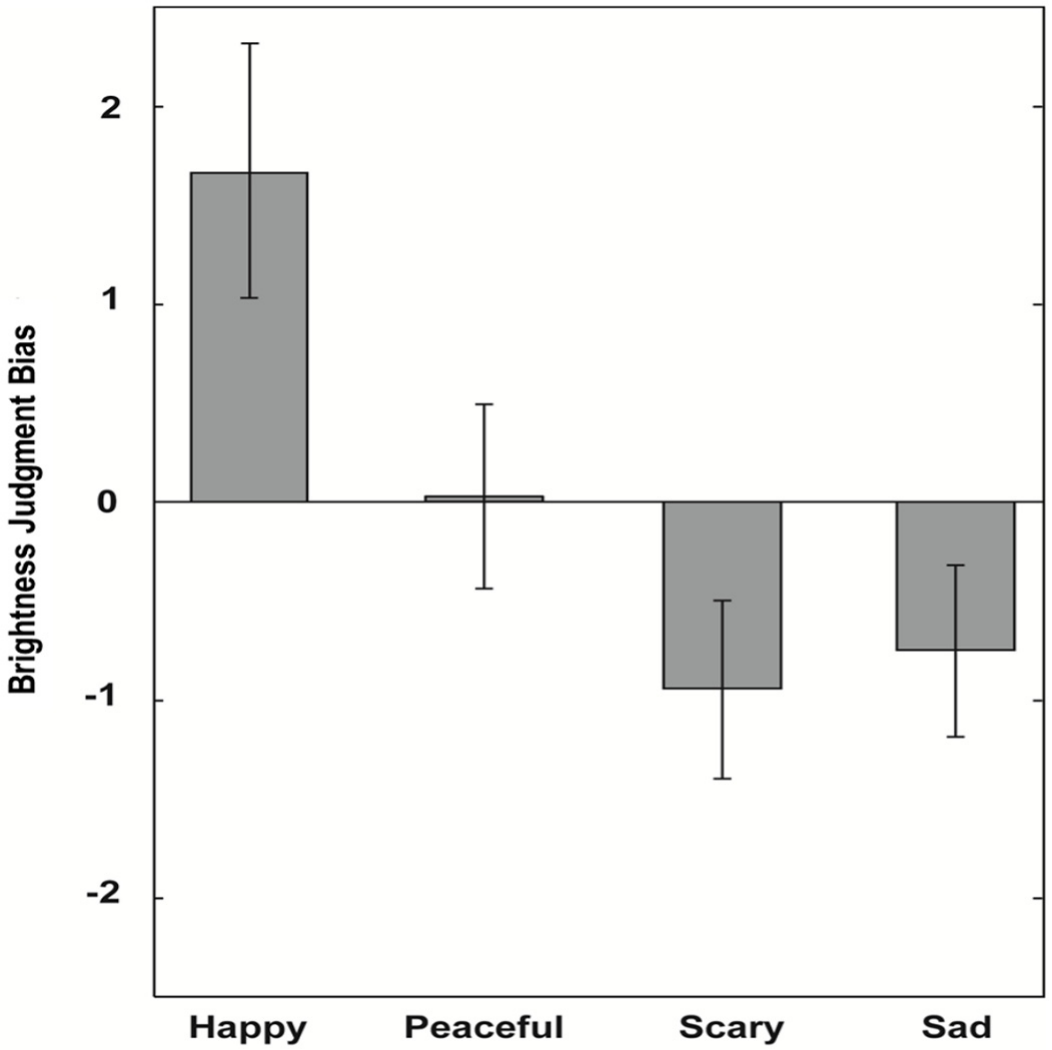
Results of Experiment 3. brightness judgment biases, separately for happy, peaceful, scary, and sad excerpts. Error bars indicate SE of the mean.

The grey square was judged as brighter following a musical prime with positive valence and darker following a musical prime with negative valence (Fig 4), as indicated by the highly significant effect of valence, *F*(1,19) = 12.12, *p* < 0.005. The effect size was large, *r* = 0.62. There was no significant main effect of arousal, *F*(1,19) = 1.29, *p* = .27, and no significant interaction between valence and arousal, *F*(1,19) = 2.19, *p* = .155.

These results confirm our earlier findings of Experiments 1 and 2, but using a substantially different paradigm. Again, the valence of a short musical prime affects the perceived brightness of a subsequently presented grey square, with positive valence biasing judgment towards brighter and negative valence biasing towards darker responses. Importantly, the results of Experiment 3 rule out any effect of memory related processes in the brightness judgment, and unambiguously show that it is the judgment of a grey square presented physically at the time of judgement that is likely to be biased. Therefore, the reported brightness judgment bias may indeed be considered as a robust effect.

## General Discussion

Across three experiments, we showed that relatively short musical excerpts could be used as effective emotional primes that cross-modally influence brightness judgment of visual stimuli. Grey squares were consistently judged as brighter after listening to music with a positive valence, as compared to music with a negative valence. This influence is consistent with the prevalent metaphorical mapping (positive = bright; negative = dark). The results of Experiment 1 confirmed our prediction that even relatively short (~10–15 s) excerpts of music can communicate emotions strong enough to bias visual judgment in a systematic fashion. The results of Experiment 2 revealed that the bias in brightness judgment does not require an active evaluation of the emotional content of the music. By applying a different experimental procedure in Experiment 3, we showed that this judgment bias is indeed a robust effect. Due to the nature of explicitly acquired judgments on visual brightness, we cannot establish whether the reported bias is a high-level conceptual effect or a low-level perceptual effect. However, the bias is consistent with metaphoric viewpoint and there are some suggestions that metaphors are not arbitrarily formed but rather grounded on low-level embodied processes [2, 32]; nevertheless, for confirming the latter perceptual account, electrophysiological study should investigate in future to find early neural components of visual processing in relation with the reported brightness bias.

Note that out of the four emotion categories (happy, peaceful, scary, and sad) studied here, musical excerpts associated with happy emotion produced the most consistent and robust brightness bias across the three experiments. Recently, Fritz and colleagues [17] showed that happy or joyful music is easier recognized as compared to scary and sad music, and also that happy emotions are universally recognized in music. If indeed happy music induces corresponding emotions in listeners more robustly and consistently than other emotions, this might explain our finding that the happy excerpts were most effective in biasing brightness judgment.

Unlike happy excerpts, the scary and sad musical excerpts were associated with most variable brightness judgment bias. This might be due to the fact that there is more confusion in recognizing scary and sad emotions in music [17]. Although the brightness bias varied over experiments, post-hoc analyses showed that the differences for scary and sad primes between Experiments 1 and 2, which allow for a straightforward comparison, were not statistically significant (Sad: *t*(38) = 1.27, *p* = .211; Scary: *t*(38) = 0, *p* = 1). This indicates that the observed variation is most likely due to chance, and does not indicate systematic difference due to, for example, the differences in experimental procedure between Experiments 1 and 2.

Although the current results suggest a clear link between musical emotion and brightness judgment, they do not necessitate that felt emotions are a crucial link in this effect, i.e. there might be a direct, unmediated association between certain acoustical or musical features and brightness perception. In general, we believe that the link between musical emotion and brightness judgment is mediated by emotion, since it is the specific combinations of acoustic and musical features that typically make emotions in music recognizable [33], not the presence of one single acoustic feature. For example, high pitch has been associated with brighter shades by both humans [30, 34, 35] and chimpanzees [30]. However, in our pool of musical excerpts, happy musical primes have comparable average pitch (midi encoding of pitch, 65.60, see Methods) to that of peaceful musical primes (62.76) and scary musical primes have comparable pitch range (53) to that of sad primes (49). Further, the acoustic brightness might conceptually prime visual brightness, and acoustic brightness of major chord is usually higher than of a minor chord. Though our happy musical primes have significantly higher acoustical brightness than of sad musical primes (*t*(1, 13) = 8.71, *p* < .01), they are not significantly different from those of peaceful musical primes (*t*(1,13) = 1.18, *p* = 0.26). What makes some music happy and other music conveying a different emotion is a certain combination of a pitch, pitch range with other parameters such as tempo, pitch variability, speed of tone attack, direction of contour, acoustic brightness etc. [33]. Therefore, simple cross-modal correspondence is unlikely to be the principal mechanism of the reported brightness judgment bias, though we do believe that future experiments with closely controlled parameters for each emotional category could offer novel insight towards a better understanding of the musical emotion induced visual judgment bias. Noteworthy to add here that as opposed to how music could influence brightness judgment, a recent study has investigated how brightness could influence music listening experience [36]: greater physiological changes are observed during listening to music in dimmer light condition than in the standard light condition.

Finally, our results need to be discussed in the wider framework of musical semantics, i.e. how music can give rise to meaning. Although it has long been disputed that music has meaning [37, 38], a growing body of recent research literature has provided evidence that musical information is perceived by human as meaningful [39], and engages neural mechanism underlying the processing of semantical information in the brain (see for a review, [40]). One of the possible mechanisms by which meaning is communicated by music is its emotional content [41, 42]. Musical emotion is fast recognized [43], and there are suggestions that this recognition almost automatically leads to the activation of concepts semantically related to the perceived emotion [44], akin to the spreading activation mechanism underlying priming [45, 46]. Cross-modal priming paradigms using music as primes have demonstrated that the N400, an event-related-potential component indexing semantic incongruity [47], is robustly elicited for mismatches in emotional meaning between music and language stimuli [44, 48–50]. We propose here that the meaning as emerged by the emotional content of a musical prime interacts with the concepts elicited metaphorically by the visual stimuli, and this interaction was found to be most robust for the happy music.

Altogether, our findings demonstrate a powerful role of musical emotion in modulating judgment of visual brightness response in a metaphor consistent fashion, the essence of which, was eloquently captured by Leonard Bernstein [51], who concluded, "… music is a totally metaphorical language."
